# IMPLEMENTATION SCIENCE IN THE DEVELOPMENT OF RECRUITMENT PROCEDURES AND MATERIALS: A CASE STUDY

**DOI:** 10.1093/geroni/igad104.3421

**Published:** 2023-12-21

**Authors:** Cameron Ulmer, Johanna Silbersack, Lauren Stratton, Sam Fazio, Sheryl Zimmerman

**Affiliations:** University of North Carolina at Chapel Hill, Chapel Hill, North Carolina, United States; University of North Carolina at Chapel Hill, Chapel Hill, North Carolina, United States; Alzheimer’s Association, Chicago, Illinois, United States; Alzheimer’s Association, Chicago, Illinois, United States; University of North Carolina at Chapel Hill, Chapel Hill, North Carolina, United States

## Abstract

Recruiting long-term care organizations, administrators, and direct-care staff to participate in research has become increasingly challenging after the pandemic due to staffing shortages, limited resources, and numerous competing priorities. In light of these challenges, it is especially important to develop recruitment procedures and materials that are fine-tuned to the participant group based on best practices. This presentation will describe the use of implementation science principles and an expert focus group in the design of recruitment procedures and materials for a NIA-funded clinical trial “Evaluating a National Person-Centered Training Program to Strengthen the Dementia Care Workforce.” The project is especially well-suited to employ and critique best practices given that recruitment is at both the organizational-level (assisted living communities) and the person-level (staff within those communities). Recruitment materials were designed in accordance with the Considering Determinants of Diffusion, Dissemination, and Implementation of Innovations in Health Service Delivery and Organization conceptual model, which emphasizes highlighting the advantages of the training, how the training fits into existing systems, the process of training, the endorsement of organizational leaders, and the transfer of the knowledge conveyed by the training into practice. Best practices from the literature were discussed with a focus group of assisted living experts to evaluate the draft procedures and materials. This presentation will share the resulting procedures and materials and explain their evolution to their final form; the process and results are widely applicable to other clinical trials and other studies. Funded by a grant from the National Institute on Aging (R01 AG079124).

